# Age-dependent acquisition of IgG antibodies to *Shigella* serotypes—a retrospective analysis of seroprevalence in Kenyan children with implications for infant vaccination

**DOI:** 10.3389/fimmu.2024.1340425

**Published:** 2024-02-01

**Authors:** Melissa C. Kapulu, Esther Muthumbi, Edward Otieno, Omar Rossi, Pietro Ferruzzi, Francesca Necchi, Alessandra Acquaviva, Laura B. Martin, Benedict Orindi, Kennedy Mwai, Hillary Kibet, Alfred Mwanzu, Godfrey M. Bigogo, Jennifer R. Verani, Cecilia Mbae, Christopher Nyundo, Charles N. Agoti, Usman Nasir Nakakana, Valentino Conti, Philip Bejon, Samuel Kariuki, J. Anthony G. Scott, Francesca Micoli, Audino Podda

**Affiliations:** ^1^ KEMRI-Wellcome Trust Programme, Kilifi, Kenya; ^2^ Centre for Tropical Medicine and Global Health, Nuffield Department of Medicine, University of Oxford, Oxford, United Kingdom; ^3^ Department of Infectious Disease Epidemiology, London School of Hygiene and Tropical Medicine, London, United Kingdom; ^4^ GSK Vaccines Institute for Global Health, Siena, Italy; ^5^ Epidemiology and Biostatistics Division, School of Public Health, University of the Witwatersrand, Johannesburg, South Africa; ^6^ Centre for Global Health Research, Kenya Medical Research Institute, Kisumu, Kenya; ^7^ Division of Global Health Protection, US Centers for Disease Control and Prevention, Nairobi, Kenya; ^8^ Centre for Microbiology Research, Kenya Medical Research Institute, Nairobi, Kenya

**Keywords:** *Shigella flexneri*, *Shigella sonnei*, sero-prevalence, serotype, Kenya, IgG antibodies

## Abstract

**Background:**

Shigellosis mainly affects children under 5 years of age living in low- and middle-income countries, who are the target population for vaccination. There are, however, limited data available to define the appropriate timing for vaccine administration in this age group. Information on antibody responses following natural infection, proxy for exposure, could help guide vaccination strategies.

**Methods:**

We undertook a retrospective analysis of antibodies to five of the most prevalent *Shigella* serotypes among children aged <5 years in Kenya. Serum samples from a cross-sectional serosurvey in three Kenyan sites (Nairobi, Siaya, and Kilifi) were analyzed by standardized ELISA to measure IgG against *Shigella sonnei* and *Shigella flexneri* 1b, 2a, 3a, and 6. We identified factors associated with seropositivity to each *Shigella* serotype, including seropositivity to other *Shigella* serotypes.

**Results:**

A total of 474 samples, one for each participant, were analyzed: Nairobi (*n* = 169), Siaya (*n* = 185), and Kilifi (*n* = 120). The median age of the participants was 13.4 months (IQR 7.0–35.6), and the male:female ratio was 1:1. Geometric mean concentrations (GMCs) for each serotype increased with age, mostly in the second year of life. The overall seroprevalence of IgG antibodies increased with age except for *S. flexneri* 6 which was high across all age subgroups. In the second year of life, there was a statistically significant increase of antibody GMCs against all five serotypes (*p* = 0.01–0.0001) and a significant increase of seroprevalence for *S. flexneri* 2a (*p* = 0.006), *S. flexneri* 3a (*p* = 0.006), and *S. sonnei* (*p* = 0.05) compared with the second part of the first year of life. Among all possible pairwise comparisons of antibody seropositivity, there was a significant association between *S. flexneri* 1b and 2a (OR = 6.75, 95% CI 3–14, *p* < 0.001) and between *S. flexneri* 1b and 3a (OR = 23.85, 95% CI 11–54, *p* < 0.001).

**Conclusion:**

Children living in low- and middle-income settings such as Kenya are exposed to *Shigella* infection starting from the first year of life and acquire serotype-specific antibodies against multiple serotypes. The data from this study suggest that *Shigella* vaccination should be targeted to infants, ideally at 6 or at least 9 months of age, to ensure children are protected in the second year of life when exposure significantly increases.

## Introduction

*Shigella* is a major cause of bacillary diarrhea, including dysentery, and is transmitted by the fecal–oral route, through ingestion of contaminated food or water. Ninety-nine percent of all cases occur in low- and middle-income countries (LMICs), and approximately 70% occur in children younger than 5 years of age (1, 2). Sixteen serotypes (all 14 *S. flexneri*, *S. sonnei*, and *S. dysenteriae* type 1) are considered to be of global importance (3), with *S. sonnei* being the most common serotype worldwide. The Global Enteric Multicenter Study (GEMS), which aimed to determine the incidence and etiology of moderate to severe diarrhea (MSD) in children aged less than 5 years in Africa and South Asia, found that i) *Shigella* is the most common cause of MSD in children aged 12–59 months; ii) attributable incidence of *Shigella* MSD is the highest in children aged 12–23 months, with the median age of cases at 20 months; and iii) approximately 72% of *Shigella* MSD cases were caused by *S. sonnei* (~24%), *S. flexneri* 1b (7.5%), *S. flexneri* 2a (~20%), *S. flexneri* 3a (~9%), and *S. flexneri 6* (11%) (2, 4, 5).

Improved hygiene and sanitation could significantly reduce the disease burden, but this is unlikely to be accomplished in the short term in most LMICs, where *Shigella* is endemic, considering the need for a large investment of resources and strong political will. Shigellosis can be treated with antibiotics; however, treatment options are increasingly limited, as resistance to commonly used antibiotics, including ciprofloxacin, is increasingly reported (3, 6, 7). In this context, given that approximately 70% of *Shigella* cases occur in children younger than 5 years of age, the development of a *Shigella* vaccine, effective against the principal disease-causing serotypes, is attractive, and based on GEMS data, its administration in early childhood would be most impactful.

Natural exposure to *Shigella* induces short-term serum IgG and secretory IgA (at the mucosal sites of infection) responses that have been shown to be serotype-specific and directed to the O-antigen portion of lipopolysaccharide (LPS) (8–11). In Kenya, prevalence studies have focused on the isolation of the bacterium from diarrheal stools obtained from various populations in different geographical locations with rates varying between 2.8% and 24% (12–14). However, the age of infection with disease-causing serotypes is still poorly defined in children. Chisenga et al. recently described the IgG and IgA antibody responses in the first year of life to *S. flexneri* 2a and *S. sonnei* in Zambian infants (8). To date, these results form the only data available on the acquisition of antibodies to infection in infants albeit to only two serotypes. Thus, there is a need to determine the concentration and the seroprevalence of specific antibodies to other serotypes of major importance for early-age exposure in order to complement epidemiological data and further guide vaccine development and vaccination implementation.

With this in mind, we undertook a retrospective multicenter seroprevalence study in Kenyan children.

## Materials and methods

### Study design, setting, and participants

This was a retrospective laboratory-based study. We used archived samples from children aged less than 5 years collected in 2015 from a cross-sectional study investigating the prevalence of fecal carriage and seroprevalence of non-typhoidal *Salmonella* at three sites in Kenya (**Supplementary Figure 1**). Volunteers were recruited from three sites in Mukuru, Nairobi County; Asembo, Siaya County; and Junju, Kilifi County (**Supplementary Figure 1**). Mukuru (referred to as Nairobi) is an urban informal settlement (slum area) in Nairobi City (15, 16); Asembo (referred to as Siaya), in Western Kenya, is a semirural setting (17) and was a Kenyan site included in the GEMS study (4, 18); while Junju (referred to as Kilifi) is a rural setting on the Kenyan Coast where children have been enrolled in a longitudinal malaria cohort from birth to the age of 15 years separate from the current study (19, 20). Participants were identified evenly across the three sites by age-stratified, simple random sampling into 0–6-month, 7–11-month, 12–23-month, 24–35-month, 36–47-month, and 48–59-month strata, taking equal proportions in each stratum. The sampling frame was based either on the most recent census data (Nairobi), population-based surveillance census data (Siaya), or demographic health surveillance data (Kilifi). Fieldworkers within these areas recruited the participants after information sharing and obtaining informed consent from the parent/guardian of the participating children.

Participants were asked to attend a clinic visit at each trial location, at enrolment where anthropometric measurements such as weight, height (length for those under 2 years of age), and mid-upper arm circumference (MUAC) were collected. In addition, a short questionnaire was administered to the parent/guardian to collect information on sociodemographic and environmental (e.g., drinking water source, washing water source, toilet type) characteristics. Data on diarrhea episodes (defined as >3 loose stools in 24 h) and febrile illness (defined as temperature >38°C) within the last 2 weeks of the enrolment visit were also collected. A point-of-care test was conducted for hemoglobin level as well as a *Plasmodium falciparum* malaria HRP-2-based rapid diagnostic test (RDT). As part of the main study, the primary sample for analysis was stool, which was prioritized for collection, whereas sera were the secondary samples collected alongside the stools; thus, not all volunteers who were enrolled had a 2-ml blood sample collected for serum separation. A subset of the stool samples from the Kilifi site underwent PCR using the TaqMan array (21), with the results for the *Shigella* PCR targets available for analysis. Serum samples from children aged less than 5 years were assayed for serotype-specific IgG to *Shigella*.

### Enzyme-linked immunosorbent assay

After collection, serum samples were stored at −80°C in the KEMRI-Wellcome Trust Biobank until 2021 when an aliquot of each was shipped to GSK Vaccine Institute for Global Health in Siena, Italy, for analysis. Anti-*Shigella* serum IgG was measured by standardized enzyme-linked immunosorbent assay (ELISA) using *S. flexneri* 6 and *S. sonnei* LPS or *S. flexneri* 1b, *S. flexneri* 2a, and *S. flexneri* 3a O-antigen (OAg) as coating antigens (at final concentrations of 15 µg/ml in PBS for *S. flexneri* 6, 0.5 µg/ml in PBS for *S. sonnei*, 2 µg/ml in carbonate buffer for *S. flexneri* 1b, 0.5 µg/mL in carbonate buffer for *S. flexneri* 2a, and 1 µg/ml in PBS for *S. flexneri* 3a, respectively) and 1:4500 dilution of goat anti-human IgG-alkaline phosphatase secondary antibody (Wholesaler, UK Sigma-Aldrich) using the methodology previously described (22). ELISA units were expressed relative to a five-parameter human antigen-specific antibody standard serum curve composed of 10 standard points and 2 blank wells (run in duplicate on each plate). One ELISA unit is defined as the reciprocal of the dilution of the standard serum that gives an absorbance value [optical density (OD) measured at 405 nm minus the OD measured at 490] equal to 1 in the assay. Standard serum to assess anti-*S. sonnei* LPS-specific antibodies was obtained by pooling sera from four individual high responders initially enrolled in a clinical trial who received two vaccinations with *S. sonnei* monocomponent 1790GAHB (23), whereas the standard sera used to assess anti-*S. flexneri*-specific OAg antibodies were obtained by pooling sera prevaccination from nine individual subjects enrolled in the clinical trial conducted in Pakistan and India (24), where *Shigella* is endemic, that were screened for response against each of the abovementioned *S. flexneri* serotypes. Anti-*S. sonnei* standard was used to determine anti-*S. sonnei*-specific antibodies in a phase 2b trial conducted with 1790GAHB (25), and the assay was fully characterized in terms of standard curve accuracy, linearity, specificity, precision, and limits of quantification; the anti-*S. sonnei* standard sera were calibrated in order to meet the definition of 1 EU as the reciprocal of the dilution giving an OD equal to 1 for this study. The primary anti-*S. flexneri* standard sera were calibrated against each coating antigen, and the assay was characterized in terms of standard curve accuracy and specificity. The limits of blanks and quantification were established.

Several QC criteria were applied on each run, particularly minimum *R* ^2^ value ≥0.96 for the 5PL curve fit to standard dilution series, maximum background <0.15 OD for *S. sonnei* coating and <0.1 for *S. flexneri* coating, minimum value of OD maximum ≥2.6, range between 0.5 OD and 2 OD for 1 EU/ml, and Control Dev <40% to the expected EU/ml both for the high and low controls. If at least one of the abovementioned criteria was not met, the entire layout was repeated under the same experimental conditions. For each sample, the EU/ml was determined as the average of EU/ml of the triplicate at each specific sera dilution if the control CV was <30%; otherwise, the sample was rerun under the same conditions. The limits of standard curve accuracy (LSCAs) were set at the nominal value of the standard at the last and the first serial dilutions, respectively, where the confidence interval of RE% with 90% probability fell within the acceptance range of [−25%; +25%]. The lower limit of quantification (LLoQ) was set as equivalent to lower LSCA multiplied by 100 (the lowest sera dilution tested in the assay). Specificity was determined by running ELISA on the samples depleted with 50 μg/ml of homologs or heterologous polysaccharides in comparison with the undepleted control. The assay was considered specific if inhibition was >80% in the presence of homologous polysaccharide and <20% with heterologous polysaccharides (either from the same species, thus *S. sonnei* for *S. flexneri* and *S. flexneri* for *S. sonnei*, and from a different species—*Salmonella* Typhimurium for all).

Anti-specific LPS/OAg IgG antibodies were summarized by age class and site as GMCs, while seropositivity was expressed as a binary variable (1 = positive; 0 = negative) and defined as an antibody concentration at least four-fold greater than the lower limit of quantification (4 * LLoQ) for each specific serotype. The LLoQ was 9 EU/ml for *S. sonnei*, 8 EU/ml for *S. flexneri* 1b, 2 EU/ml for *S. flexneri* 2a, 11 EU/ml for *S. flexneri* 3a, and 2 EU/ml *S. flexneri* 6.

### Variables

Our outcome variable of interest was IgG antibodies against *Shigella* measured by ELISA, assessed for each sample collected from each subject, classified as seropositive or seronegative as defined above. More specifically, as a population-level summary, we looked at seroprevalence (based on the percent of samples seropositive) and at the magnitude of IgG antibodies (as reflected by antibody GMC). Our explanatory variables for seropositivity to *Shigella* serotypes included sociodemographic characteristics, water source, toilet type, drinking water type, and serotypes. Sociodemographic characteristics for this study included age (in months), sex, diarrhea, and fever and were summarized using frequencies with anthropometric measures. Anthropometric measures were defined as follows: underweight [weight-for-age *Z*-score (WAZ) <−2]; wasting [weight-for-height *Z*-score (WHZ) <−2], and stunting [height-for-age *Z*-score (HAZ) <−2] based on the WHO Child Growth Standards (26). Anemia was defined by a hemoglobin level of less than 10 g/dl. In addition, data on anti-*Salmonella* Enteritidis and *Salmonella* Typhimurium IgG concentrations from the parent study were made available and classified as seropositive or seronegative to determine their association with *Shigella* serotype-specific antibodies. TaqMan array PCR data (only available for the Kilifi site), specific for *S. sonnei* and *S. flexneri* 1b, 2a, 3a, and 6, were used to further determine their relationship with the antibody concentrations.

### Data management and statistical analysis

The dataset was checked for missing values and completeness and exported to R version 4.0.3 (R Core Team 2019) and Stata version 15 (Stata Corp., College Station, TX, USA) for analysis. We used descriptive statistics for sociodemographic characteristics. We presented the continuous variables using median and interquartile ranges (IQRs), while categorical variables were presented using frequency and percentages. We used the Kruskal–Wallis test to determine the statistically significant difference between the medians of the continuous variables and the independent groups (study sites). We used the chi-square test to assess the association between categorical variables. Participants were grouped and analyzed into six age categories: 0–6 months, 7–11 months, 12–23 months, 24–35 months, 36–47 months, and 48–59 months. *Shigella* serotype-specific antibodies were presented together with the 95% confidence interval across age groups and by site. The non-parametric test of trend was used to compare the association between sites and the ranks across ordered age groups. We visualized the prevalence of the IgG antibody by age using a bar plot and the associated 95% Agresti–Coull confidence intervals, violin plots for the distribution of IgG antibodies by age, and heatmaps for seroprevalence by age and site. Pearson correlation was used to test the association among the respective serotype-specific antibody responses.

The chi-square test for trend was used to compare the association between seropositivity and age groups. Non-parametric one-way analysis of variance (ANOVA) test was used to determine the association between serotype antibody concentrations and age groups.

We used univariable and multivariable logistic regression models to investigate factors associated with *Shigella* serotype-specific antibodies. For each antigen, the site was checked as a potential effect modifier for the association between age and seropositivity. The presence of effect modification was determined using a likelihood ratio test (LRT). To determine the explanatory variables to be included in the final models (one for each *Shigella* serotype), we used LRT. We reported both the crude and adjusted odds ratios with their corresponding 95% confidence intervals (CIs). All tests were considered significant at *p <*0.05 with no adjustment for multiple comparisons.

## Results

### Study population characteristics

A total of 474 participants (169 from Nairobi, 185 from Siaya, and 120 from Kilifi) with a median age of 13.4 months (IQR 7.0–35.6) had their samples analyzed (**Table 1**). There were no age or sex differences across the sites.

**Table 1:** Characteristics of the study population.

	Nairobi		Siaya		Kilifi		Total		*p*
	*n* = 169		*n* = 185		*n* = 120		*N* = 474		
	*n*	%	*n*	%	*n*	%	*n*	%	
Age category (months)									
0–6	39	23.1	40	21.6	28	23.3	107	22.6	0.12
7–11	34	20.1	48	25.9	32	26.7	114	24.1	
12–23	33	19.5	22	11.9	13	10.8	68	14.3	
24–35	27	16.0	30	16.2	11	9.2	68	14.3	
36–47	25	14.8	23	12.4	24	20.0	72	15.2	
48–59	11	6.5	22	11.9	12	10.0	45	9.5	
Sex									
Female	79	46.7	102	55.1	56	46.7	237	50.0	0.28
Fever	25	14.8	68	36.8	19	15.8	112	23.6	**<0.001**
Diarrhea	33	19.5	20	10.8	11	9.2	64	13.5	**0.02**
Stunting	38	22.5	32	17.3	27	22.5	97	20.5	0.3
Wasting	13	7.7	5	2.7	10	8.3	28	5.9	**0.05**
Underweight	14	8.3	13	7.0	18	15.0	45	9.5	**0.03**
Anemia	18	10.7	15	8.1	39	32.5	72	15.2	**<0.001**
Illness history in family members									
Fever	12	7.1	67	36.2	14	11.7	93	19.6	**<0.001**
Diarrhea	13	7.7	14	7.6	7	5.8	34	7.2	0.89
	Median	IQR	Median	IQR	Median	IQR	Median	IQR	
Weight (kg)	9.6	7.4–12.2	10.0	8.6–13.1	9.3	7.4–12.4	9.8	7.9–12.4	**0.04**
Height (cm)	75.0	66.3–87	75.5	68.1–92	76.0	67.5–92.8	75.0	67–90	0.14
MUAC (cm)	14.4	13.1–15.5	16.0	14.2–18	14.0	13.1–15	14.9	13.5–16	**0.0001**
HB (g/dl)	11.1	10.4–12.1	11.6	10.5–12.7	10.6	9.2–11.2	11.0	10.1–12.1	**0.0001**

MUAC, middle-upper arm circumference; HB, hemoglobin; kg, kilogram; cm, centimeter; p-diarrhea, denotes whether the participant had diarrhea at the time of sampling; m-diarrhea, denotes whether a member of the household had diarrhea at the time of sampling; p-febrile, indicates whether the participant had a fever at the time of sampling; and m-febrile, denotes whether a member of the household had a fever at the time of sampling. Data are presented as either median and (IQR, interquartile range) or n and (%). Significance considered at *p* <0.05. In bold are statistically significant p-values.

As shown in **Table 1**, diarrhea and fever in the preceding 2 weeks were reported among 13.5% (64/474) and 23.6% (112/474) participants, respectively, and diarrhea and fever were reported among 7.2% (34/474) and 19.6% (93/474) family members, respectively. At the time of sampling, 15.2% (72/474) of the participants had anemia, 20.5% (97/474) were stunted, 5.9% (28/474) were wasted, and 9.5% (45/474) were underweight. Several site-specific differences were observed: diarrhea episodes were more commonly reported in Nairobi (*p* = 0.02); both participants and household members had more frequent fever episodes in Siaya (*p* < 0.001); and anemia (*p* < 0.001), wasting (*p* = 0.050), and being underweight (*p* = 0.03) were more common in Kilifi.

### Seroprevalence of IgG antibodies against *Shigella*

**Figure 1:**
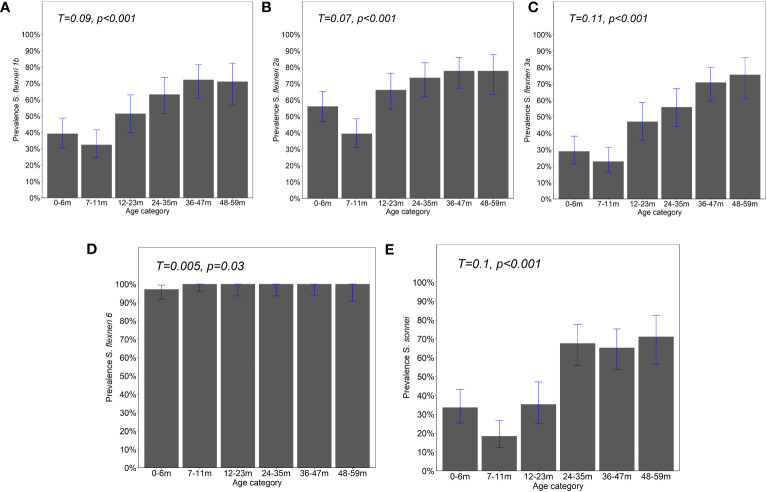
Prevalence of IgG antibody responses by age. IgG antibody responses to the OAg of **(A)** *Shigella flexneri* 1b, **(B)** *S. flexneri* 2a, **(C)** *S. flexneri* 3a, **(D)** *S. flexneri* 6, and **(E)** *S. sonnei* were measured by ELISA. Bar plot showing the proportion of positive individuals based on the participants with responses above the four-fold lower limit of quantification (LLoQ). Shown is the overall prevalence with 95% Agresti–Coull confidence intervals. Chi-square test—test for trend. Significance considered at *p <*0.05.

**Figure 2:**
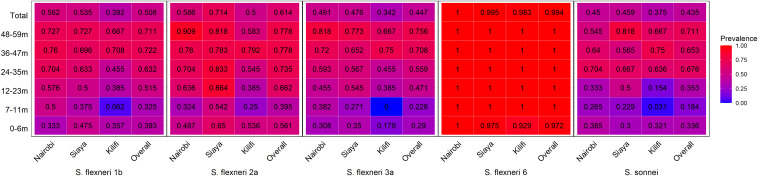
Seroprevalence heatmap by age and site. Serum antibody prevalence by age against the different serotypes was determined by ELISA. The heatmap represents all the three sites and the overall prevalence with proportion indicated in each individual box. The gradient of the heatmap from blue to red indicates the lowest to the highest proportions, respectively.

The observed seroprevalence of antibodies to *S. flexneri* 1b, *S. flexneri* 2a, *S. flexneri* 3a, and *S. sonnei* was 50.8%, 61.4%, 44.7%, and 43.5%, respectively, showing a relationship with age. However, no age-related difference was observed for *S. flexneri* 6 (100% across all ages and sites apart from 0 to 6 months for Kilifi and Siaya at 92.9% and 97.5%, respectively) (**Table 2** and **Figure 1**). The overall age-related difference was significant for *S. flexneri* 2a (*p* = 0.01) and *S. sonnei* (*p* = 0.01), for which a U-shaped relationship was observed where the seroprevalence was 56.1% and 33.6% at 0–6 months and decreased to 39.5% and 18.4% in the 7–12-month group, respectively, for *S. flexneri* 2a and *S. sonnei.* Seroprevalence increased in the subsequent age groups with peaks observed above the 36-month and 48-month age groups for *S. flexneri* 2a and *S. sonnei*, respectively. Seroprevalence was the lowest among the 7–11-month age groups against *S. flexneri* 1b, *S. flexneri* 2a, *S. flexneri* 3a, and *S. sonnei*. Prevalence against *S. flexneri* 1b, *S. flexneri* 2a, *S. flexneri* 3a, and *S. sonnei* was ≥70% by the age of 59 months. A significant prevalence increase was shown in the second year of life compared with the first year of life for *S. flexneri* 2a (*p* = 0.006), *S. flexneri* 3a (*p* = 0.006), and *S. sonnei* (*p* = 0.05). Except for *S. flexneri* 6, there was no evidence that the site modifies the association between age and antigen seropositivity (**Figure 2**).

**Table 2:** Seroprevalence of antibodies by age and site.

Serotype	Site	0–6 months	7–11 months	12–23 months	24–35 months	36–47 months	48–59 months	Total	*p*
*Shigella flexneri* 1b	Nairobi	34 (21–49)	50 (34–66)	58 (41–73)	70 (51–84)	76 (56–89)	73 (43–91)	56 (49-64)	**0.006**
	Siaya	48 (33–63)	38 (25–52)	50 (31–69)	63 (46–78)	70 (49–85)	73 (52–87)	54 (46–61)	**0.03**
	Kilifi	36 (21–54)	6 (7–21)	39 (18–65)	46 (21–72)	71 (51–85)	67 (39–86)	39 (31–48)	**<0.001**
	Overall	39 (31–49)	33 (25–42)	52 (40–63)	63 (51–74)	72 (61–81)	71 (57–82)	51 (46–55)	0.29
*Shigella flexneri* 2a	Nairobi	49 (34–64)	32 (19–49)	64 (47–78)	70 (51–84)	76 (56–89)	91 (60–100)	59 (51–66)	**<0.001**
	Siaya	65 (50–78)	54 (40–67)	86 (66–96)	83 (66–93)	78 (58–91)	82 (61–93)	71 (64–77)	**0.02**
	Kilifi	54 (36–71)	25 (13–42)	39 (18–65)	55 (28–79)	79 (59–91)	58 (32–81)	50 (41–59)	**0.004**
	Overall	56 (47–65)	40 (31–49)	66 (54–76)	74 (62–83)	78 (67–86)	78 (64–88)	61 (57–66)	**0.01**
*Shigella flexneri* 3a	Nairobi	31 (19–47)	38 (24–55)	46 (30–62)	59 (41–76)	72 (52–86)	82 (51–96)	49 (42–57)	**0.003**
	Siaya	35 (22–51)	27 (17–41)	55 (35–73)	57 (39–73)	65 (45–81)	77 (56–90)	48 (41–55)	**<0.001**
	Kilifi	18 (7–36)	0 (0–13)	39 (18–65)	46 (21–72)	75 (55–88)	67 (39–86)	34 (26–43)	**<0.001**
	Overall	29 (21–38)	23 (16–31)	47 (36–59)	56 (44–67)	71(59–80)	76 (61–86)	45 (40–49)	0.30
*Shigella flexneri* 6	Nairobi	100 (89–100)	100 (88–100)	100 (88–100)	100 (85–100)	100 (84–100)	100 (70–100)	100 (97–100)	–
	Siaya	98 (86–100)	100 (91–100)	100 (83–100)	100 (87–100)	100 (83–100)	100 (83–100)	100 (97–100)	0.60
	Kilifi	93 (76–99)	100 (87–100)	100 (73–100)	100 (70–100)	100 (84–100)	100 (72–100)	98 (94–100)	0.25
	Overall	97 (92–99)	100 (96–100)	100 (94–100)	100 (94–100)	100 (94–100)	100 (91–100)	99 (98–100)	0.07
*Shigella sonnei*	Nairobi	39 (25–54)	27 (14–43)	33 (20–51)	70 (51–84)	64 (44–80)	55 (28–79)	45 (38–53)	**0.003**
	Siaya	30 (18–46)	23 (13–37)	50 (31–70)	67 (49–81)	57 (37–74)	82 (61–93)	46 (39–53)	**<0.001**
	Kilifi	32 (18–51)	3 (0–17)	15 (3–44)	64 (35–85)	75 (55–88)	67 (39–86)	38 (29–46)	**<0.001**
	Overall	34 (25–43)	18 (12–27)	35 (25–47)	68 (56–78)	65 (54–75)	71 (57–82)	44 (39–48)	**0.01**

Data are presented as percent seroprevalence (%) with 95% confidence intervals in parenthesis. Significance considered at *p* <0.05. In bold are statistically significant p-values.

### Magnitude and breadth of the *Shigella*-specific IgG antibody response

**Figure 3:**
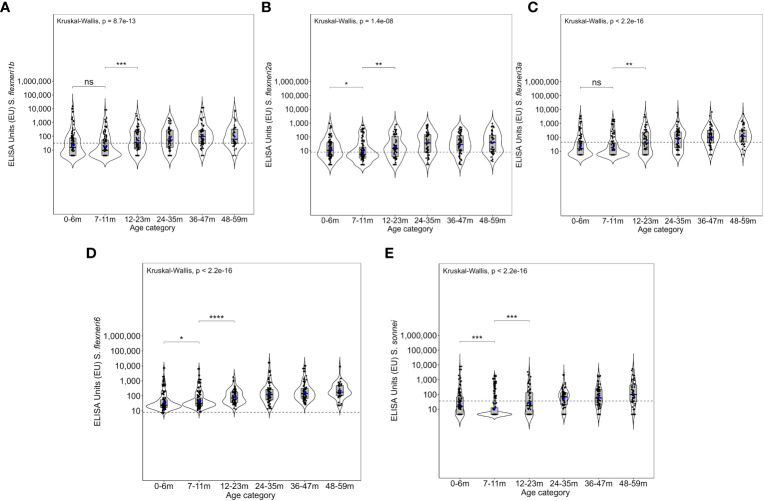
Overall magnitude of IgG antibodies by age. Violin plots of the anti-OAg IgG ELISA units (EU) against **(A)** *Shigella flexneri* 1b, **(B)** *S. flexneri* 2a, **(C)** *S. flexneri* 3a, **(D)** *S. flexneri* 6, and **(E)** *S. sonnei*. Indicated within each violin plot are boxplots with the median (dark solid line) and the lower and upper quartiles with the geometric mean concentration represented in blue dots. Each individual is represented by each individual black closed circle. The dotted line represents the seroresponse cutoff of the antibodies. The Kruskal–Wallis test with multiple comparison adjustment was used. A test for all pairwise comparisons between age groups and ELISA unit concentrations was conducted. Significance is denoted by an asterisk with significance considered at *p <*0.05, where *p < 0.05, **p < 0.01, ***p < 0.001, and ****p < 0.0001. ns, not significant.

**Figure 4:**
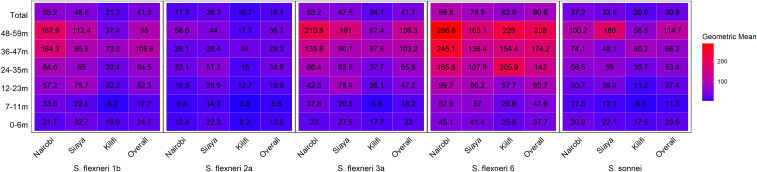
Geometric mean concentration heatmap by age and site. Serum antibody geometric mean concentration by age against the different serotypes was determined by ELISA. The heatmap represents all the three sites and the overall with the geomean indicated in each individual box. The gradient of the map from blue to red represents the lowest to the highest concentrations, respectively.

Overall, the magnitude of the antibody response increased with age (**Figure 3** and **Supplementary Figure 2**). The geometric mean concentration of the antibodies in the 12–23-month age groups was significantly higher compared with the 7–11 months for all five serotypes (Kruskal–Wallis, *p* = 0.01–0.0001). A significant difference was also observed for *S. flexneri* 2a, *S. flexneri* 6, and *S. sonnei* in the antibody concentrations between 0–6-month- and 7–11-month-old children, whereas there was no difference for *S. flexneri* 1b [24.7 (95% CI 17–36) vs. 17.7 (95% CI 13–25)] and *S. flexneri* 3a [23 (95% CI 16–32) vs. 18.2 (95% CI 13–25)]. Concerning the site, the patterns were similar across the three sites with no difference in the geometric mean antibodies against *S. flexneri* 2a between those 0–6 months and 7–11 months or those 7–11 months and 12–23 months old across all the three sites (**Figure 4**) with low antibody levels detected despite the high seroprevalence (**Figure 1**). Interestingly, all antibodies against *S. flexneri* 6 were above the seropositivity cutoff (**Figure 3D**).

**Figure 5:**
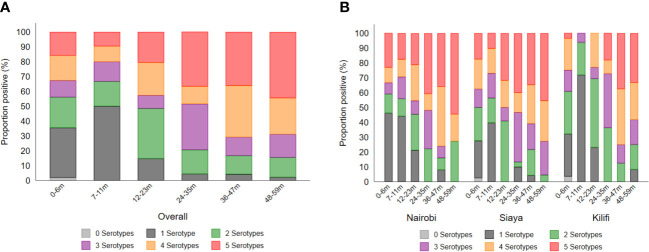
Breadth of antibody responses by **(A)** age and **(B)** site. The breadth of antibody response was defined as the number (1–5) of serotypes to which individuals had antibody concentrations above the seroresponse cutoff. Each segment of the stacked bar represents the percentage of seropositive individuals with responses to the number (*n*) of serotypes.

The breadth of antibody response by age and site is shown in **Figure 5** as well as the correlation of the antibody response among the various *Shigella* serotypes, *Salmonella* Typhimurium, and *Salmonella* Enteritidis (**Supplementary Figure 3**). Overall, exposure to multiple serotypes (*n* = 2 to 5) increased from ~50% at 7–11 months to ~85% by the age of 12–23 months (**Figure 5A**). Overall, in the 48–59-month age group, approximately 50% of children had antibodies to all serotypes, and approximately 50%, 45%, and 35% of participants in Nairobi, Siaya, and Kilifi, respectively, had antibodies to all five serotypes (**Figure 5B**). All antibody responses across the five *Shigella* serotypes were significantly positively correlated. Importantly, a strong association was observed between the presence of *S. flexneri* 1b and *S. flexneri* 3a (*ρ* = 0.864, *p <* 0.001) (**Supplementary Figure 3**).

In the multivariable analysis, being seropositive against *S. flexneri* 3a increased the odds of being *S. flexneri* 1b positive by 23.85 (95% CI 11–54, *p* < 0.001) (**Supplementary Table 4**). We further observed a positive correlation between the responses to the five *Shigella* serotypes with antibody responses against both *Salmonella* Enteritidis and *Salmonella* Typhimurium (**Supplementary Figure 3**). In a subset of subjects, analysis of stools by PCR did not show any relationship between the presence of *Shigella* in stool samples and antibodies (**Supplementary Table 3**). PCR positivity for this analysis was only 9.8%. All the other covariates tested after adjustment had no effect on seropositivity.

## Discussion

In this retrospective, multicenter study, we have shown that young children in Kenya are exposed in the first year of life to more than one serotype and subsequently develop antibodies against all five serotypes tested. We observed in the first 6 months of life the presence of antibodies suggestive of maternal origin against all five tested serotypes. Samples collected in children aged 7–11 months showed lower antibody levels, both in terms of seroprevalence and geometric mean concentrations, likely due to the waning of maternal antibodies. This observed decrease in antibodies in the first year of life followed by exposure to *Shigella* during early childhood is consistent with the findings of Chisenga et al. for *S. flexneri* 2a and *S. sonnei* in their cohort study in Zambia (8), and we extend those findings to additional serotypes.

We observed that after the first year of life, there is a significant increase of naturally acquired antibodies in addition to an age-dependent increase in the magnitude of antibody concentration for all five serotypes. Except for *S. flexneri* 6, the same age-related trend is true also for seroprevalence. Seroprevalence for *S. flexneri* 6 (99.4%) was significantly higher than that of the other four serotypes, which ranged from 43.5% to 61.4%. This could be due to earlier or more intense exposure to *S. flexneri* 6 in Kenya, intrinsic characteristics of *S. flexneri* 6, or cross-reactivity with *Escherichia coli* due to similarities of their respective O antigens (27). Should early-onset infection be the explanation, potential implications on vaccine implementation (i.e., the optimum age for vaccination) should be evaluated.

Analysis of the breadth of response to specific serotypes by age showed that ~50% of children aged 7–11 months had responses to more than one serotype, rising to ~85% during the second year of age.

Taken together, the results of this study show that the acquisition of antibody responses to *S. flexneri* 1b, *S. flexneri* 3a, *S. flexneri* 6, *S. flexneri* 2a, and *S. sonnei* in Kenya, regardless of geodemographic classification of the sites, occurs already in the first year of life, significantly increases during the second year of life, and further rises thereafter until the age of 5 years.

Particularly important, for programmatic considerations, is the significant increase of the antibody response, in terms of antibody concentrations, seroprevalence, and breadth of response, that we have observed during the second year of life. This is in agreement with the results from the GEMS study showing that the attributable incidence of qPCR-derived *Shigella*/EIEC MSD episodes per 100 child-years is 7.0 in the second year of life, compared with 2.0 in the first year of life and 2.3 in the 24–59-month age group, and that the median age of *Shigella*/EIEC MSD cases across the GEMS sites is 20 months (2, 5), which suggests a decline after the second year of life when increased exposure to *Shigella* leads to the generation of protective antibodies. Our findings therefore support the need for the development of multivalent broad-coverage vaccines including serotypes assessed in this seroprevalence study and, additionally, that vaccination should be targeted, and ideally completed, within the first year of life in order to provide the greatest impact on the burden of shigellosis in children of endemic regions. While vaccines against multiple serotypes are being developed using various platform technologies (28, 29), it is important that their suitability for administration very early in life (i.e., at 6 or 9 months) is tested in clinical trials.

Only for *S. sonnei* has a serological correlate of protection among children 2–4 years been proposed at 1:1,072 titer for anti-*S. sonnei* LPS serum IgG (30), which corresponds to 236 EU/ml in the ELISA assay used for the present study. However, it should be noted that this threshold was derived based on 10% subset sera samples from 2- to 4-year-old participants in an efficacy trial in Israel between 2003 and 2008 which did not show statistically significant vaccine efficacy in infants and toddlers younger than 2 years of age (31). More information on correlates of protection will hopefully come from vaccine field efficacy trials allowing seroprotection rates for the various serotypes to be defined.

In the absence of serological correlates of protection, it is important to evaluate whether the naturally acquired or vaccine-induced serotype-specific antibodies show complement-mediated serum bactericidal activity (SBA) *in vitro*. The functionality of vaccine-induced responses has been shown against *S. sonnei*- or *S. flexneri* 2a-based vaccines in adults (32, 33) and in an animal model of a multivalent vaccine (34). More recently, it was shown that maternal antibodies have functional activity (35) and that unvaccinated Kenyan adults have high SBA titers against *S. sonnei* (36). By contrast, no data are available about the functional activity of anti-*Shigella* antibodies generated in children and, thus, the requirement for sera from this study to address this question. Likewise, as we observed a high prevalence of antibodies against *S. flexneri* 2a (61.4%, 95% CI 56.9–65.7), but low GMCs (18.1, 95% CI 15.4-21.3), compared with all the other serotypes, evaluation of whether these anti-*S. flexneri* 2a low-level antibodies do show bactericidal killing is warranted.

We did not observe any association between water, sanitation, and hygiene (WaSH)-related variables and seropositivity (**Supplementary Tables 4–7**). Considering the WaSH importance for reducing the burden of diarrheal diseases, the lack of association in this study is difficult to interpret and could be due to the low infecting dose of *Shigella*, which may require a very high standard of WaSH (37). In addition, as indicated above, there was limited power for subgroup analysis, and further evidence is required to conclusively rule out potential factors that may predict seropositivity.

There are some limitations to this study. Firstly, the cross-sectional design does not allow us to evaluate the seroprevalence in the same children over time. However, this methodological limitation has been mitigated in this study by age stratification of the population of interest (i.e., children aged <5 years) so that we can still identify differences in the seroprevalence of different age subgroups. Secondly, the limited sample size of the study, particularly when data are stratified by site, reduces the power to make robust conclusions on site comparisons. Thirdly, while we have been able to examine seroprevalence by age for the key disease-causing serotypes, we are unable to calculate the seroprotection rate and define whether seropositive children are protected or not due to lack of accepted serological correlates of protection for *Shigella*. Fourthly, only Kenyan data have been produced in this study, and we cannot fully rule out that our results may not be applicable in different contexts, although consistency with antibody responses in the cohort study in Zambia (where 2 out of the 5 serotypes were analysed) and with the epidemiological results of the GEMS study provides reassurance on the validity of the results, at least for low- and middle-income countries of Africa and Asia.

In conclusion, the findings from this study provide evidence that in the first year of life, a high proportion of Kenyan children, from three geodemographically different regions of the country, are already exposed to *Shigella* (with antibody response used as a proxy for exposure), and exposure increases with age against all five serotypes measured. Young infants appear to have maternal antibodies which wane, and the lowest seroprevalence is in the 7–11-month age group. The significant increase of antibody concentrations and seroprevalence during the second year of age, consistent with epidemiological data from the GEMS study, provides additional arguments for the need to develop highly effective multivalent vaccines against *Shigella* to be administered by the end of the first year of life (ideally at 6 or at least 9 months of age), to offer protection before the peak of the disease starts in the second year of life.

## Data availability statement

The datasets presented in this study can be found in online repositories. The names of the repository/repositories and accession number(s) can be found below: Harvard Dataverse; https://doi.org/10.7910/DVN/F5AV5G.

## Ethics statement

Ethical approval for the conduct of this study was provided by the Kenya Medical Research Institute Scientific Ethics Review Unit (KEMRI/SERU/CGMR-C/034/3221). Written informed consent to 364 participate in this study was provided by the all-participants’ legal guardian/next of kin.

## Author contributions

MK: Writing – original draft. EM: Writing – review & editing. EO: Writing – review & editing. OR: Writing – review & editing. PF: Writing – review & editing. FN: Writing – review & editing. AA: Writing – review & editing. LM: Writing – review & editing. BO: Writing – review & editing. KM: Writing – review & editing. HK: Writing – review & editing. AM: Writing – review & editing. GB: Writing – review & editing. JV: Writing – review & editing. CM: Writing – review & editing. CN: Writing – review & editing. CA: Writing – review & editing. UN: Writing – review & editing. VC: Writing – review & editing. PB: Writing – review & editing. SK: Writing – review & editing. JS: Writing – review & editing. FM: Writing – review & editing. AP: Writing – original draft.
