# Supplementary material for: Age-dependent acquisition of IgG antibodies to Shigella serotypes—a retrospective analysis of seroprevalence in Kenyan children with implications for infant vaccination
Source: Front Immunol. 2024 Feb 1;15:1340425. doi: 10.3389/fimmu.2024.1340425 (PMC10867106; doi:10.3389/fimmu.2024.1340425)
Supplement: Supplementary file 1 [file DataSheet_1.pdf]

Supplementary Material

1 Supplementary Figures and Tables

1.1 Supplementary Figures

Supplementary Figure 1. Location map of the study sites in Kenya. Kilifi site is depicted in red, Siaya in orange, and Nairobi in green.

Supplementary Figure 2. Scatter plots showing IgG antibody responses with age. Blue lines indicate mean values with the shaded grey areas representing 95% confidence intervals (A) *S. flexneri* 1b; (B) *S. flexneri* 2a; (C) *S. flexneri* 3a; (D) *S. flexneri* 6; and (E) *S. sonnei*. Each dot represents an individual child. Dotted line across the y-axis represents the sero-positivity cut-off for each respective serotype.

Supplementary Figure 3. Spearman correlations of antibody response metrics. Correlation matrix of IgG antibody responses against the 5 *Shigella* serotypes, *Salmonella* Typimurium, and *Salmonella* Enteritidis. Heat map showing the spearman correlation coefficient. Shown in each individual box is the spearman rank correlation coefficient with the blue to red colour gradient representing a weak to strong correlation respectively. Significance is denoted by * with significance considered at $p < 0.05$ where *$p < 0.05$, **$p < 0.01$, and ***$p < 0.001$.

1.2 Supplementary Tables

Supplementary Table 1: Prevalence comparisons by age within each site.

Serotype	Site	0-6 vs 12-23 *p*	0-6 vs 7-11 *p*	0-6 vs 24-35 *p*	0-6 vs 36-47 *p*	0-6 vs 48-59 *p*	7-11 vs 12-23 *p*	7-11 vs 24-35 *p*	7-11 vs 36-47 *p*	7-11 vs 48-59 *p*	12-23 vs 24-35 *p*	12-23 vs 36-47 *p*	12-23 vs 48-59 *p*	24-35 vs 36-47 *p*	24-35 vs 48-59 *p*	36-47 vs 48-59 *p*
S. *flexneri* 1b	Nairobi	0.47	0.88	0.04	0.01	0.25	0.88	0.88	0.47	0.88	0.88	0.88	0.88	0.88	0.88	0.88
	Siaya	0.85	0.85	0.85	0.85	0.66	0.85	0.34	0.16	0.09	0.85	0.85	0.85	0.85	0.85	0.85
	Kilifi	0.87	**0.05**	0.87	0.12	0.57	0.08	**0.03**	**<0.001**	**<0.001**	0.87	0.5	0.87	0.87	0.87	0.87
	Overall	0.67	0.77	**0.02**	**<0.001**	**0.004**	0.09	**0.0006**	**<0.001**	**<0.001**	0.77	0.09	0.26	0.77	0.77	0.9
S. *flexneri* 2a	Nairobi	0.65	0.65	0.65	0.3	0.14	0.12	0.04	**0.01**	**0.01**	0.65	0.65	0.65	0.65	0.65	0.65
	Siaya	0.78	0.89	0.88	0.89	0.89	0.13	0.13	0.6	0.34	0.89	0.89	0.89	0.89	0.89	0.89
	Kilifi	0.96	0.3	0.96	0.58	0.96	0.96	0.71	**0.0009**	0.45	0.96	0.19	0.96	0.96	0.96	0.96
	Overall	0.92	0.12	0.16	0.03	0.12	0.006	**<0.001**	**<0.001**	**<0.001**	1	0.88	0.92	1	1	1
S. *flexneri* 3a	Nairobi	0.55	0.55	0.23	0.02	**0.03**	0.55	0.55	0.13	0.14	0.55	0.39	0.36	0.55	0.55	0.55
	Siaya	0.81	0.88	0.64	0.23	**0.02**	0.26	0.11	**0.03**	**0.001**	0.88	0.88	0.81	0.88	0.81	0.88
	Kilifi	0.63	0.11	0.52	**0.0005**	**0.03**	**0.002**	**0.0006**	**<0.001**	**<0.001**	0.73	0.23	0.63	0.52	0.73	0.73
	Overall	0.09	0.58	0.004	**<0.001**	**<0.001**	**0.006**	**<0.001**	**<0.001**	**<0.001**	0.58	**0.03**	**0.02**	0.26	0.17	0.58
S. *flexneri* 6	Nairobi	-	-	-	-	-	-	-	-	-	-	-	-	-	-	-
	Siaya	0.45	0.45	0.45	0.45	0.45	-	-	-	-	-	-	-	-	-	-
	Kilifi	0.36	0.36	0.36	0.36	0.36	-	-	-	-	-	-	-	-	-	-
	Overall	0.26	0.26	0.26	0.26	0.26	-	-	-	-	-	-	-	-	-	-
S. *sonnei*	Nairobi	0.65	0.65	0.13	0.46	0.65	0.65	0.009	0.06	0.65	0.06	0.22	0.65	0.65	0.65	0.65
	Siaya	0.66	0.66	0.03	0.31	**0.001**	0.23	**0.002**	0.06	**<0.001**	0.66	0.66	0.23	0.66	0.66	0.47
	Kilifi	0.88	**0.03**	0.43	**0.02**	0.3	0.68	**<0.001**	**<0.001**	**<0.001**	0.12	**0.006**	0.08	0.88	0.88	0.88
	Overall	0.82	**0.05**	**<0.001**	**<0.001**	**<0.001**	**0.05**	**<0.001**	**<0.001**	**<0.001**	**0.001**	**0.003**	**0.002**	0.82	0.82	0.82

Significance considered at $p<0.05$. In bold are statistically significant *p* values.

Supplementary Table 2: Geometric mean concentrations by age and site.

Serotype	Site	0-6 months	7-11 months	12-23 months	24-35 months	36-47 months	48-59 months	Total
S. *flexneri* 1b	Nairobi	21.7(10.9 - 43.2)	33.6(16 - 70.5)	57.2(29.6 - 110.4)	84.6(39.9 - 179.6)	164.3(67.9 - 397.4)	187.9(42.5 - 829.9)	55.2(39.8 - 76.6)
	Siaya	32.7(16.9 - 63)	22.6(13.3 - 38.5)	75.7(30.9 - 185.4)	65(33.5 - 126.2)	95.8(44.8 - 204.9)	112.4(49.6 - 254.5)	48.6(36.5 - 64.5)
	Kilifi	19.9(10.7 - 37.2)	6.2(4.5 - 8.4)	22.2(6.7 - 73.5)	32.4(8.5 - 123.3)	73.2(40.1 - 133.5)	37.4(23.1 - 60.4)	21.3(15.8 - 28.7)
	Overall	24.7(16.9 - 36)	17.7(12.5 - 24.8)	52.3(32.6 - 83.8)	64.5(41.1 - 101.2)	105.6(69 - 161.6)	95(55.3 - 163.2)	41.3(34.5 - 49.4)
S. *flexneri* 2a	Nairobi	12.4(7.2 - 21.3)	8.8(4.4 - 17.4)	16.5(8.4 - 32.4)	32.1(15.3 - 67.4)	26.1(13 - 52.5)	56.6(18.7 - 171.9)	17.5(13.2 - 23.3)
	Siaya	22.3(12.8 - 38.9)	14.3(9 - 22.8)	29.5(14.2 - 61.2)	51.2(27.4 - 95.4)	28.4(13.7 - 58.9)	44(18.7 - 103.2)	26.3(20.4 - 33.8)
	Kilifi	8.2(4.7 - 14.1)	3.9(2.5 - 6.1)	12.7(3.4 - 47.7)	15(4 - 55.9)	34(16.2 - 71.5)	17.7(8 - 39.5)	10.7(7.8 - 14.6)
	Overall	13.8(10 - 19)	8.6(6.3 - 11.8)	18.9(12 - 29.9)	34.9(22.4 - 54.2)	29.3(19.7 - 43.6)	36.7(21.8 - 61.7)	18.1(15.4 - 21.3)
S. *flexneri* 3a	Nairobi	23(12.5 - 42.3)	37.8(18.5 - 76.9)	42.5(20.8 - 87)	86.4(43.4 - 172)	135.8(69.4 - 265.7)	210.8(57.4 - 773.8)	53.2(39.3 - 71.9)
	Siaya	27.5(15.2 - 49.7)	20.9(13.3 - 33)	78.4(33 - 186.5)	62.8(33.8 - 116.8)	90.7(43.2 - 190.6)	161(83.5 - 310.6)	47.5(36.4 - 61.9)
	Kilifi	17.7(10 - 31.6)	6.8(5.7 - 8)	26.1(9.9 - 68.8)	37.7(10.8 - 131.8)	87.6(50.3 - 152.4)	67.4(31.7 - 143.4)	24.1(18.1 - 32.1)
	Overall	23(16.4 - 32.2)	18.2(13.4 - 24.7)	47.2(29.5 - 75.5)	65.6(43.3 - 99.6)	103.2(71.7 - 148.5)	136.3(85.2 - 218.2)	41.7(35.2 - 49.2)
S. *flexneri* 6	Nairobi	45.1(28.2 - 72)	57.9(36.9 - 90.9)	99.7(67.7 - 146.9)	165.6(94 - 291.5)	245.1(132.2 - 454.3)	286.8(106.2 - 774.1)	98.8(78.7 - 123.9)
	Siaya	41.4(26.3 - 65)	57(38.1 - 85.3)	86.2(52.6 - 141.3)	107.9(60.3 - 193.1)	136.4(75.6 - 246)	168.1(99.1 - 285)	78.5(63.8 - 96.7)
	Kilifi	25.8(18.9 - 35.4)	29.6(23.5 - 37.2)	57.7(36.3 - 91.5)	205.9(105.4 - 402.3)	154.4(105.3 - 226.4)	229(141.5 - 370.6)	62.9(50.7 - 77.9)
	Overall	37.7(29.4 - 48.5)	47.6(38 - 59.7)	85.7(66.4 - 110.5)	142(100.3 - 201)	174.2(128.7 - 235.9)	208(146.4 - 295.6)	80.6(70.9 - 91.5)
S. *sonnei*	Nairobi	30.9(15.7 - 60.7)	17.5(8.2 - 37.7)	30.7(14.5 - 64.7)	56.5(34.3 - 92.8)	74.1(33.8 - 162.4)	100.2(21.7 - 462.9)	37.2(27.3 - 50.7)
	Siaya	22.1(12.2 - 40.3)	12.1(7.7 - 18.9)	39.4(16 - 97.4)	59(32.7 - 106.3)	48.1(27.8 - 83.1)	180(78.6 - 412.6)	33.6(25.6 - 44.1)
	Kilifi	17.5(10.3 - 29.8)	6.5(4.3 - 9.6)	11.2(5.1 - 24.8)	35.7(14.3 - 89.3)	80.2(38.8 - 165.7)	56.8(25.4 - 127.1)	20.8(15.5 - 28)
	Overall	23.5(16.5 - 33.4)	11.3(8.3 - 15.5)	27.4(17 - 44.4)	53.4(37.9 - 75.3)	66.2(44.9 - 97.6)	114.7(65.4 - 201.1)	30.9(26 - 36.6)

Data are presented as geometric mean concentrations (ELISA Units, EU) with 95% confidence intervals in parenthesis.

Supplementary Table 3: Taqman PCR array positivity in relation to serotype antibody positivity for Kilifi

		PCR				
ELISA		Negative	Positive	Total	Chi *p*	RR
S. *flexneri* 1b	Negative	21	2	23	0.80	1.12
	Positive	16	2	18		
S. *flexneri* 2a	Negative	18	2	20	0.96	0.97
	Positive	19	2	21		
S. *flexneri* 3a	Negative	25	2	27	0.48	1.54
	Positive	12	2	14		
S. *sonnei*	Negative	24	2	26	0.58	1.42
	Positive	13	2	15		
S. flexneri 6	Positive	37	4	41		
	Total	37	4	41		

Supplementary Table 4: Logistic regression model predicting factors associated with *S. flexneri* 1b seropositivity

**Covariate**		Unadjusted OR (95%CI)	*p*	Adjusted OR (95%CI)	*p*
Age in months	0-6m	ref		ref	
	7-11m	0.74 (0.42-1.29)	0.29	1.14 (0.35-3.76)	0.82
	12-23m	1.64 (0.88-3.03)	0.11	0.98 (0.26-3.62)	0.99
	24-35m	2.66 (1.42-4.98)	**0.002**	0.69 (0.18-2.62)	0.59
	36-47m	4.02 (2.11-7.67)	**<0.001**	0.53 (0.12-2.23)	0.39
	48-59m	3.80 (1.79-8.08)	**<0.001**	0.39 (0.07-2.16)	0.28
Location	Nairobi	ref		ref	
	Kilifi	0.50 (0.31-0.80)	**0.004**	0.96 (0.14-6.59)	0.97
	Siaya	0.89 (0.58-1.36)	0.61	1.48 (0.23-9.17)	0.67
Sex	Male	ref			
	Female	0.81 (0.56-1.16)	0.26		
S. Typhimurium	Negative	ref		ref	
	Positive	2.42 (1.41-4.15)	**0.001**	1.24 (0.31-4.90)	0.76
S. Enteritidis	Negative	ref		ref	
	Positive	3.79 (2.01-7.13)	**<0.001**	2.08 (0.52-8.18)	0.29
S. *flexneri* 2a	Negative	ref		ref	
	Positive	6.62 (4.35-10.06)	**<0.001**	6.75 (3.19-14.26)	**<0.001**
S. *flexneri* 3a	Negative	ref		ref	
	Positive	38.56 (22.34-66.56)	**<0.001**	23.85 (10.62-53.53)	**<0.001**
S. *sonnei*	Negative	ref		ref	
	Positive	5.66 (3.78-8.45)	**<0.001**	1.83 (0.83-4.03)	0.13
S. *flexneri* 6	Negative	ref			
	Positive	**			
Salmonella Breadth	0 serotypes	ref		ref	
	1 serotype	2.69 (0.93-7.74)	**0.07**	0.51 (0.12-2.04)	0.34
	2 serotypes	6.11 (2.27-16.44)	<0.001	**	
Drinking Water Source	Piped water	ref		ref	
	Protected sources	1.20 (0.71-2.04)	0.49	1.57 (0.38-6.35)	0.53
	Unprotected sources	1.84 (1.04-3.25)	**0.03**	1.81 (0.54-5.98)	0.33

**Covariate**		Unadjusted OR (95%CI)	*p*	Adjusted OR (95%CI)	*p*
Washing Water Source	Piped water	ref			
	Protected sources	1.19 (0.65-2.19)	0.55		
	Unprotected sources	1.15 (0.76-1.75)	0.50		
Toilet Type	Modern/Flush toilet	ref			
	Latrine	0.73 (0.35-1.50)	0.40		
	Others	1.15 (0.35-3.73)	0.81		
Participant Febrile	No	ref			
	Yes	1.14 (0.74-1.75)	0.53		
Participant Diarrhoea	No	ref			
	Yes	0.88 (0.52-1.50)	0.66		
Malnourished	No	ref			
	Yes	1.38 (0.51-3.71)	0.51		
Member Febrile	No	ref			
	Yes	0.87 (0.55-1.38)	0.57		
Member Diarrhoea	No	ref			
	Yes	1.23 (0.61-2.49)	0.55		
MUAC		1.00 (0.97-1.02)	0.83		
Height		1.03 (1.02-1.04)	**<0.001**	1.01 (0.97-1.04)	0.47
Weight		1.10 (1.04-1.16)	**<0.001**	0.98 (0.91-1.06)	0.71
Stunting	No	ref			
	Yes	0.85 (0.54-1.34)	0.50		
Wasting	No	ref			
	Yes	1.48 (0.67-3.23)	0.32		
Anaemic	No	ref		ref	
	Yes	0.64 (0.38-1.08)	0.10	0.41 (0.14-1.22)	0.11
Underweight	No	ref			
	Yes	1.07 (0.58-1.99)	0.81		
Household Member		1.03 (0.94-1.13)	0.48		
Floor Type	Concrete/Tiles	ref		ref	
	Dirt	0.75 (0.35-1.57)	0.45	1.15 (0.18-6.99)	0.88

Covariate		Unadjusted OR (95%CI)	*p*	Adjusted OR (95%CI)	*p*
	Dung	0.98 (0.61-1.59)	0.97	0.52 (0.12-2.20)	0.38
	Sand	0.62 (0.37-1.03)	0.07	1.61 (0.31-8.33)	0.57
	Other	0.28 (0.02-2.77)	0.28	**	
Medication	None	ref			
	Antibiotics	0.73 (0.44-1.21)	0.23		
	Other Prescription Drugs	1.61 (0.84-3.08)	0.14		
	Non-prescription Drugs	1.07 (0.68-1.70)	0.74		
Shared Toilet	Owned	ref			
	Shared	0.73 (0.47-1.12)	0.16		
Rear Animals	No	ref		ref	
	Yes	0.67 (0.46-0.97)	**0.04**	0.46 (0.11-1.88)	0.28
HB		1.12 (1.00-1.25)	**0.05**	0.98 (0.83-1.16)	0.86

Supplementary Table 5: Logistic regression model predicting factors associated with *S. flexneri* 2a seropositivity

		Unadjusted		Adjusted	
**Covariate**		OR (95%CI)	*p*	OR (95%CI)	*p*
Age in months	0-6m	ref		ref	
	7-11m	0.51 (0.29-0.87)	0.01	0.47 (0.18-1.26)	0.14
	12-23m	1.53 (0.81-2.88)	0.18	1.08 (0.37-3.12)	0.87
	24-35m	2.17 (1.12-4.21)	**0.02**	1.22 (0.40-3.66)	0.72
	36-47m	2.74 (1.39-5.38)	**0.003**	1.41 (0.42-4.70)	0.58
	48-59m	2.74 (1.23-6.10)	**0.01**	2.46 (0.56-10.82)	0.23
Location	Nairobi	ref		ref	
	Kilifi	0.70 (0.44-1.13)	0.15	0.72 (0.20-2.56)	0.62
	Siaya	1.76 (1.13-2.73)	**0.01**	1.05 (0.28-3.82)	0.94
Sex	Male	ref			
	Female	0.83 (0.57-1.21)	0.34		
S. Typhimurium	Negative	ref		ref	
	Positive	1.95 (1.16-3.27)	**0.01**	1.21 (0.44-3.29)	0.70
S. Enteritidis	Negative	ref		ref	
	Positive	2.00 (1.15-3.49)	**0.01**	1.80 (0.65-4.94)	0.25
S. *flexneri* 1b	Negative	ref		ref	
	Positive	6.62 (4.35-10.06)	**<0.001**	7.18 (3.33-15.47)	**<0.001**
S. *flexneri* 3a	Negative	ref		ref	
	Positive	4.51 (2.98-6.81)	**<0.001**	0.93 (0.41-2.11)	0.88
S. *sonnei*	Negative	ref		ref	
	Positive	3.48 (2.32-5.22)	**<0.001**	1.40 (0.71-2.76)	0.32
S. *flexneri* 6	Negative	ref			
	Positive	**			
Salmonella Breadth	0 serotypes	ref		ref	
	1 Serotype	2.23 (0.93-5.37)	0.07	1.82 (0.69-4.78)	0.22
	2 Serotypes	3.33 (1.49-7.45)	**0.003**	**	

Covariate		Unadjusted OR (95%CI)	*p*	Adjusted OR (95%CI)	*p*
Drinking Water Source	Piped water	ref		ref	
	Protected sources	1.18 (0.68-2.04)	0.54	0.52 (0.12-2.13)	0.37
	Unprotected sources	2.89 (1.47-5.64)	**0.001**	3.29 (0.76-14.19)	0.11
Washing Water Source	Piped water	ref		ref	
	Protected sources	1.19 (0.64-2.21)	0.58	1.51 (0.35-6.48)	0.57
	Unprotected sources	1.64 (1.05-2.55)	0.03	0.46 (0.13-1.60)	0.23
Toilet Type	Modern/Flush toilet	ref			
	Latrine	0.90 (0.43-1.89)	0.80		
	Others	0.89 (0.27-2.93)	0.86		
Participant Febrile	No	ref		ref	
	Yes	1.78 (1.12-2.83)	**0.01**	1.75 (0.76-4.03)	0.18
Participant Diarrhoea	No	ref		ref	
	Yes	1.22 (0.70-2.13)	0.47	1.34 (0.59-3.06)	0.47
Malnourished	No	ref			
	Yes	1.52 (0.52-4.39)	0.44		
Member Febrile	No	ref		ref	
	Yes	1.58 (0.97-2.59)	0.06	0.50 (0.21-1.16)	0.11
Member Diarrhoea	No	ref			
	Yes	0.68 (0.33-1.37)	0.29		
MUAC		1.00 (0.97-1.02)	0.96		
Height		1.02 (1.01-1.03)	**<0.001**	1.00 (0.97-1.02)	0.97
Weight		1.09 (1.03-1.16)	**<0.001**	0.99 (0.93-1.05)	0.79
Stunting	No	ref			
	Yes	0.73 (0.46-1.15)	0.18		
Wasting	No	ref			
	Yes	0.62 (0.28-1.33)	0.22		
Anaemic	No	ref		ref	

**Covariate**		Unadjusted OR (95%CI)	*p*	Adjusted OR (95%CI)	*p*
	Yes	0.92 (0.54-1.55)	0.76	1.51 (0.62-3.69)	0.36
Underweight	No	ref			
	Yes	0.68 (0.37-1.27)	0.23		
Household Member		1.01 (0.91-1.11)	0.82		
Floor Type	Concrete/Tiles	ref		ref	
	Dirt	0.80 (0.38-1.70)	0.57	1.60 (0.35-7.30)	0.54
	Dung	1.85 (1.09-3.15)	**0.02**	2.78 (0.83-9.29)	0.10
	Sand	0.58 (0.35-0.96)	**0.04**	0.73 (0.23-2.36)	0.61
	Other	1.88 (0.19-18.39)	0.59	0.91 (0.03-23.16)	0.96
Medication	None	ref		ref	
	Antibiotics	1.00 (0.59-1.67)	0.99	1.99 (0.79-4.99)	0.14
	Other Prescription Drugs	1.96 (0.96-4.00)	0.06	1.89 (0.57-6.25)	0.29
	Non-prescription Drugs	0.93 (0.58-1.48)	0.78	1.23 (0.53-2.84)	0.62
Shared Toilet	Owned	ref		ref	
	Shared	0.52 (0.32-0.84)	**0.01**	0.47 (0.20-1.10)	0.09
Rear animals	No	ref			
	Yes	1.20 (0.83-1.75)	0.32		
HB		1.05 (0.95-1.15)	0.29	0.96 (0.83-1.11)	0.64

Supplementary Table 6: Logistic regression model predicting factors associated with *S. flexneri* 3a seropositivity

Covariate		Unadjusted OR (95%CI)	*p*	Adjusted OR (95%CI)	*p*
Age in months	0-6m	ref		ref	
	7-11m	0.72 (0.39-1.32)	0.30	0.80 (0.21-3.04)	0.75
	12-23m	2.17 (1.15-4.10)	0.02	2.33 (0.59-9.22)	0.23
	24-35m	3.10 (1.64-5.86)	**<0.001**	1.24 (0.29-5.23)	0.77
	36-47m	5.95 (3.08-11.49)	**<0.001**	2.82 (0.50-15.94)	0.24
	48-59m	7.57 (3.41-16.82)	**<0.001**	2.89 (0.38-22.04)	0.30
Location	Nairobi	ref		ref	
	Kilifi	0.53 (0.33-0.87)	**0.01**	4.16 (0.62-27.66)	0.14
	Siaya	0.94 (0.61-1.42)	0.77	5.57 (0.98-31.58)	**0.05**
Sex	Male	ref			
	Female	1.02 (0.71-1.47)	0.90		
S. Typhimurium	Negative	ref		ref	
	Positive	2.18 (1.25-3.78)	**0.01**	0.59 (0.15-2.24)	0.44
S. Enteritidis	Negative	ref		ref	
	Positive	2.86 (1.52-5.39)	**0.001**	1.68 (0.44-6.42)	0.45
S. *flexneri* 1b	Negative	ref		ref	
	Positive	38.56 (22.34-66.56)	**<0.001**	21.73 (9.95-47.48)	**<0.001**
S. *flexneri* 2a	Negative	ref		ref	
	Positive	4.51 (2.98-6.81)	**<0.001**	1.03 (0.45-2.34)	0.93
S. *sonnei*	Negative	ref		ref	
	Positive	7.78 (5.15-11.74)	**<0.001**	3.99 (1.86-8.57)	**<0.001**
S. *flexneri* 6	Negative	ref			
	Positive	**			
Salmonella Breadth	0 serotypes	ref		ref	
	1 Serotype	2.90 (0.92-9.12)	**0.07**	1.85 (0.49-6.93)	0.36
	2 Serotypes	6.06 (2.06-17.84)	**0.001**		
Drinking Water Source	Piped water	ref		ref	
	Protected sources	1.07 (0.62-1.82)	0.80	0.79 (0.19-3.21)	0.74

**Covariate**		Unadjusted OR (95%CI)	*p*	Adjusted OR (95%CI)	*p*
	Unprotected sources	2.05 (1.17-3.59)	**0.01**	1.41 (0.43-4.59)	0.56
Washing Water Source	Piped water	ref			
	Protected sources	0.95 (0.52-1.75)	0.89		
	Unprotected sources	0.98 (0.64-1.49)	0.93		
Toilet Type	Modern/Flush toilet	ref			
	Latrine	1.27 (0.61-2.63)	0.51		
	Others	1.23 (0.38-3.93)	0.73		
Participant Febrile	No	ref			
	Yes	1.03 (0.67-1.59)	0.86		
Participant Diarrhoea	No	ref			
	Yes	0.88 (0.51-1.50)	0.65		
Malnourished	No	ref			
	Yes	1.79 (0.67-4.80)	0.24		
Member Febrile	No	ref			
	Yes	1.07 (0.67-1.69)	0.76		
Member Diarrhoea	No	ref			
	Yes	1.42 (0.70-2.86)	0.33		
MUAC		1.00 (0.97-1.03)	0.68		
Height		1.04 (1.03-1.05)	**<0.001**	1.04 (0.98-1.09)	0.14
Weight		1.14 (1.08-1.21)	**<0.001**	0.93 (0.85-1.03)	0.19
Stunting	No	ref			
	Yes	1.00 (0.64-1.57)	0.99		
Wasting	No	ref			
	Yes	1.66 (0.76-3.60)	0.20		
Anaemic	No	ref			
	Yes	0.87 (0.52-1.47)	0.63		
Underweight	No	ref			
Underweight	Yes	1.29 (0.69-2.38)	0.42		
Household Member		1.03 (0.94-1.13)	0.43		

Covariate		Unadjusted OR (95%CI)	*p*	Adjusted OR (95%CI)	*p*
Floor Type	Concrete/Tiles	ref		ref	
	Dirt	0.74 (0.35-1.58)	0.45	0.96 (0.15-6.14)	0.97
	Dung	0.98 (0.61-1.58)	0.95	0.76 (0.18-3.20)	0.72
	Sand	0.62 (0.37-1.03)	0.07	0.58 (0.11-2.97)	0.52
	Other	0.36 (0.03-3.56)	0.39	**	
Medication	None	ref			
	Antibiotics	0.87 (0.52-1.45)	0.61		
	Other Prescription Drugs	1.50 (0.80-2.83)	0.20		
	Non-prescription Drugs	1.06 (0.67-1.69)	0.78		
Shared Toilet	Owned	ref			
	Shared	0.85 (0.55-1.32)	0.49		
Rear animals	No	ref		ref	
	Yes	0.64 (0.44-0.92)	**0.02**	0.23 (0.06-0.87)	**0.03**
HB		1.13 (1.00-1.28)	**0.04**	1.00 (0.85-1.17)	0.98

**Denotes no group variation within the variables. Significance considered at $p<0.05$. In bold are statistically significant *p* values.

Supplementary Table 7: Logistic regression model predicting factors associated with *S. sonnei* seropositivity

Covariate		Unadjusted OR (95%CI)	*p*	Adjusted OR (95%CI)	*p*
Age in months	0-6m	ref		ref	
	7-11m	0.44 (0.23-0.82)	0.01	0.47 (0.17-1.24)	0.13
	12-23m	1.07 (0.56-2.03)	0.82	0.60 (0.22-1.64)	0.33
	24-35m	4.12 (2.15-7.87)	**<0.001**	4.57 (1.63-12.81)	**0.003**
	36-47m	3.70 (1.97-6.95)	**<0.001**	2.91 (0.96-8.77)	0.06
	48-59m	4.85 (2.27-10.37)	**<0.001**	2.90 (0.77-10.80)	0.11
Location	Nairobi	ref		ref	
	Kilifi	0.73 (0.45-1.18)	0.21	0.68 (0.32-1.47)	0.34
	Siaya	1.04 (0.68-1.58)	0.85	0.78 (0.36-1.66)	0.53
Sex	Male	ref			
	Female	1.04 (0.72-1.50)	0.82		
S. Typhimurium	Negative	ref		ref	
	Positive	2.59 (1.46-4.60)	**0.001**	2.67 (0.96-7.36)	0.06
S. Enteritidis	Negative	Ref		ref	
	Positive	2.01 (1.10-3.66)	**0.02**	0.72 (0.25-2.05)	0.55
S. flexneri 1b	Negative	ref		ref	
	Positive	5.66 (3.78-8.45)	**<0.001**	1.91 (0.91-4.00)	0.08
S. flexneri 2a	Negative	ref		ref	
	Positive	3.48 (2.32-5.22)	**<0.001**	1.44 (0.77-2.71)	0.25
S. flexneri 3a	Negative	ref		ref	
	Positive	7.78 (5.15-11.74)	**<0.001**	3.03 (1.47-6.24)	**0.003**
S. flexneri 6	Negative	ref			
	Positive	1.54 (0.13-17.11)	0.72		
Salmonella Breadth	0 serotypes	ref		ref	
	1 Serotype	2.11 (0.73-6.12)	0.17	1.43 (0.52-3.90)	0.48
	2 Serotypes	4.34 (1.61-11.69)	**0.003**		
Drinking Water Source	Piped water	ref		ref	

Covariate		Unadjusted OR (95%CI)	*p*	Adjusted OR (95%CI)	*p*
	Protected sources	1.24 (0.73-2.11)	0.42	2.22 (0.85-5.79)	0.10
	Unprotected sources	1.40 (0.80-2.43)	0.23	0.60 (0.22-1.63)	0.32
Washing Water Source	Piped water	ref			
	Protected sources	1.39 (0.76-2.55)	0.28		
	Unprotected sources	0.86 (0.56-1.31)	0.49		
Toilet Type	Modern/Flush toilet	ref			
	Latrine	1.04 (0.51-2.14)	0.90		
	Others	1.08 (0.34-3.45)	0.89		
Participant Febrile	No	ref			
	Yes	1.22 (0.79-1.88)	0.35		
Participant Diarrhoea	No	ref			
	Yes	0.87 (0.50-1.49)	0.61		
Malnourished	No	ref			
	Yes	1.16 (0.43-3.06)	0.76		
Member Febrile	No	ref			
	Yes	1.35 (0.85-2.13)	0.20		
Member Diarrhoea	No	ref			
	Yes	0.90 (0.44-1.83)	0.77		
MUAC		1.02 (0.98-1.07)	0.24		
Height		1.02 (1.01-1.04)	**<0.001**	0.98 (0.96-1.01)	0.33
Weight		1.10 (1.04-1.16)	**<0.001**	1.00 (0.94-1.07)	0.78
Stunting	No	ref			
	Yes	1.26 (0.81-1.98)	0.30		
Wasting*	No	ref			
	Yes	2.08 (0.95-4.56)	0.07		
Anaemic	No	ref			
	Yes	0.72 (0.42-1.22)	0.22		
Underweight	No	ref		ref	
	Yes	2.06 (1.10-3.86)	**0.02**	1.77 (0.65-4.84)	0.26
Household Member		0.97 (0.88-1.06)	0.57		

**Covariate**		Unadjusted OR (95%CI)	*p*	Adjusted OR (95%CI)	*p*
Floor Type	Concrete/Tiles	ref			
	Dirt	0.77 (0.36-1.66)	0.52		
	Dung	1.16 (0.72-1.87)	0.53		
	Sand	0.94 (0.57-1.56)	0.82		
	Other	0.43 (0.04-4.21)	0.47		
Medication	None	ref			
	Antibiotics	0.90 (0.54-1.51)	0.72		
	Other Prescription Drugs	1.32 (0.70-2.48)	0.38		
	Non-prescription Drugs	1.03 (0.65-1.64)	0.88		
Shared Toilet	Owned	ref			
	Shared	0.92 (0.60-1.42)	0.73		
Rear animals	No	ref			
	Yes	0.98 (0.68-1.42)	0.95		
HB		1.09 (0.98-1.22)	0.11	1.06 (0.92-1.21)	0.39

*Excluded from the multivariable model due to strong relationship with underweight. **Denotes no group variation within the variables. Significance considered at $p<0.05$. In bold are statistically significant *p* values.
