# Supplementary figures and images for: Age-dependent acquisition of IgG antibodies to Shigella serotypes—a retrospective analysis of seroprevalence in Kenyan children with implications for infant vaccination
Source: Front Immunol. 2024 Feb 1;15:1340425. doi: 10.3389/fimmu.2024.1340425 (PMC10867106; doi:10.3389/fimmu.2024.1340425)

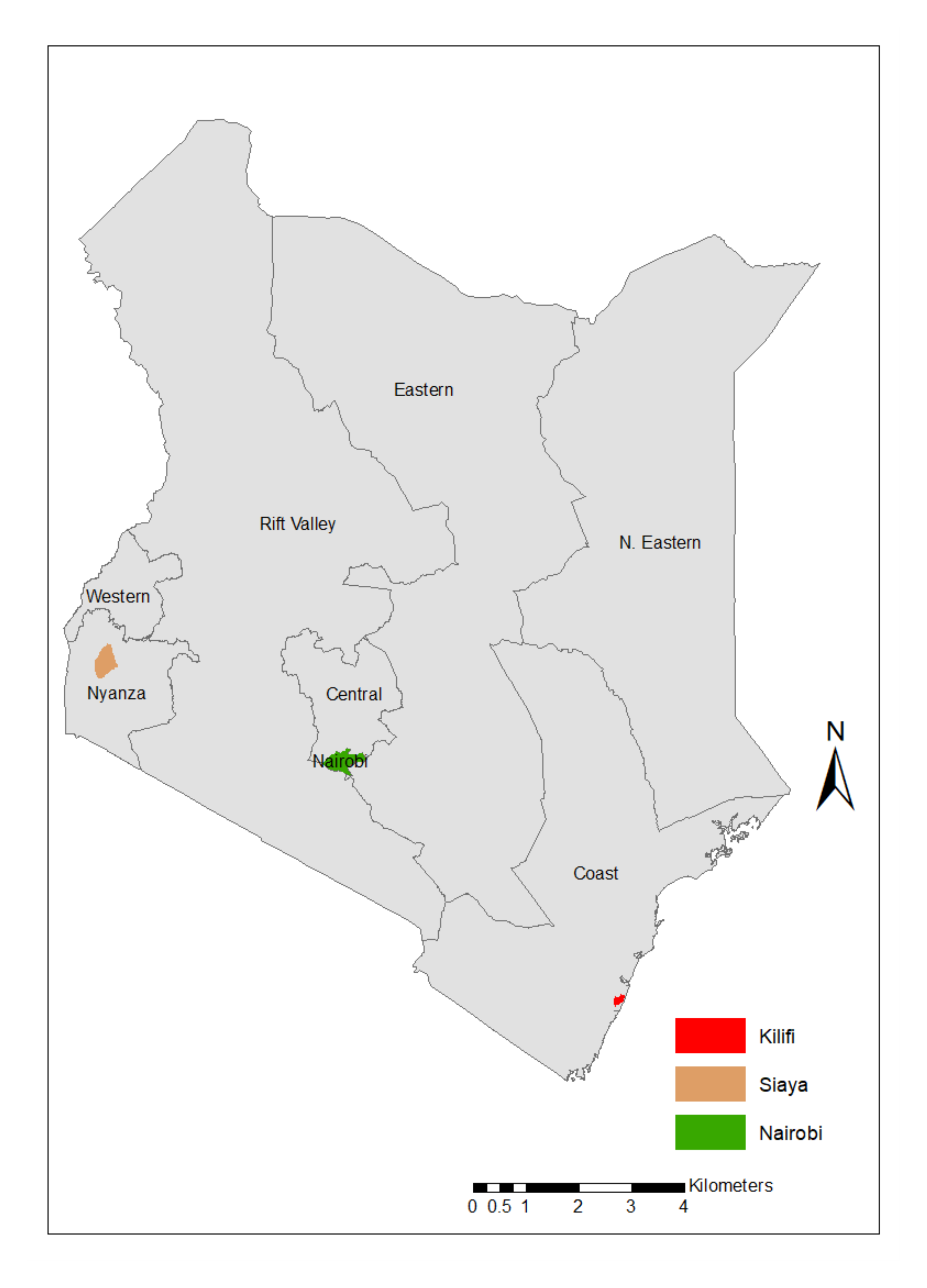

Supplement: Supplementary file 2 [file Image_1.tif]

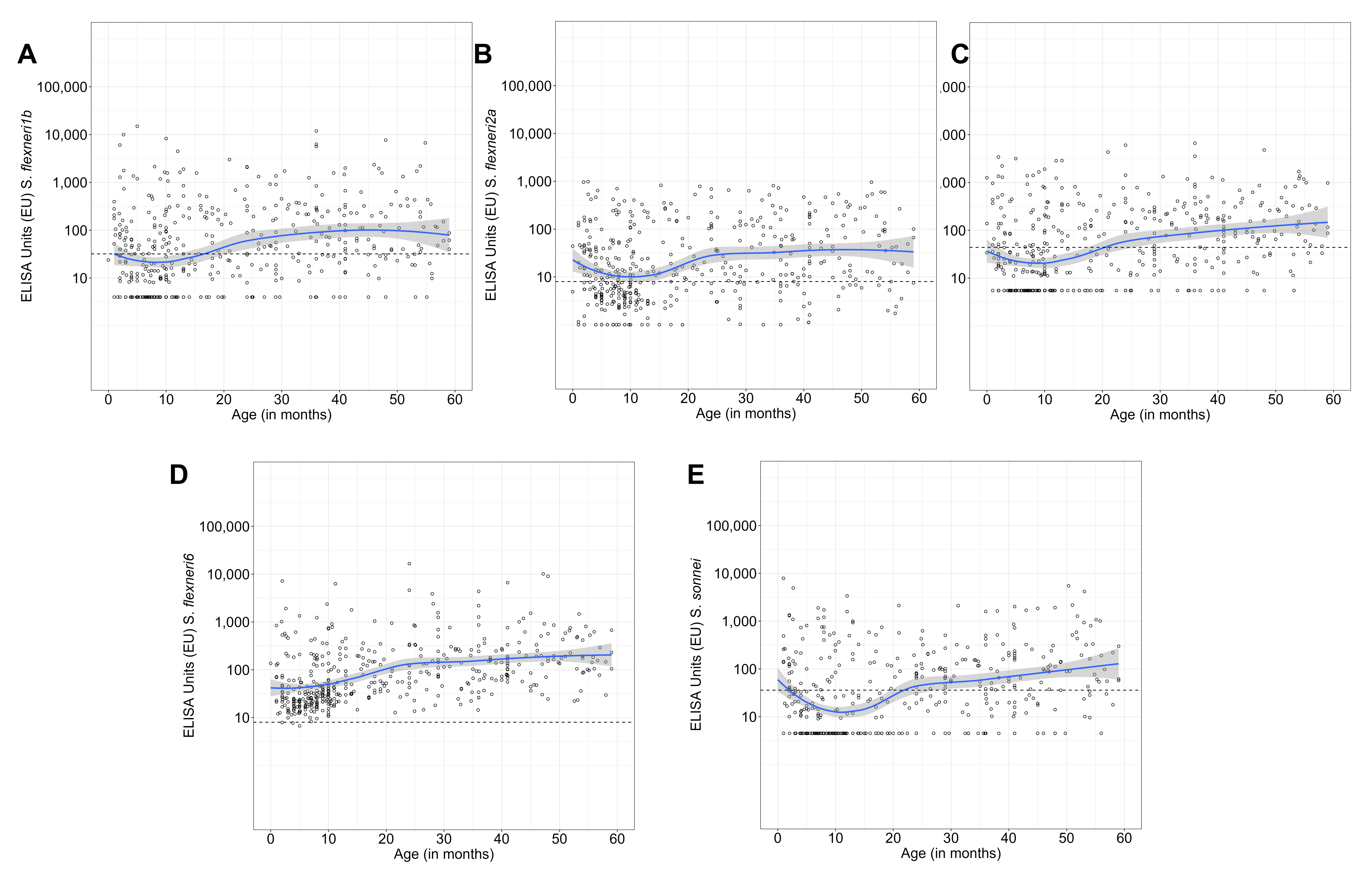

Supplement: Supplementary file 3 [file Image_2.tif]

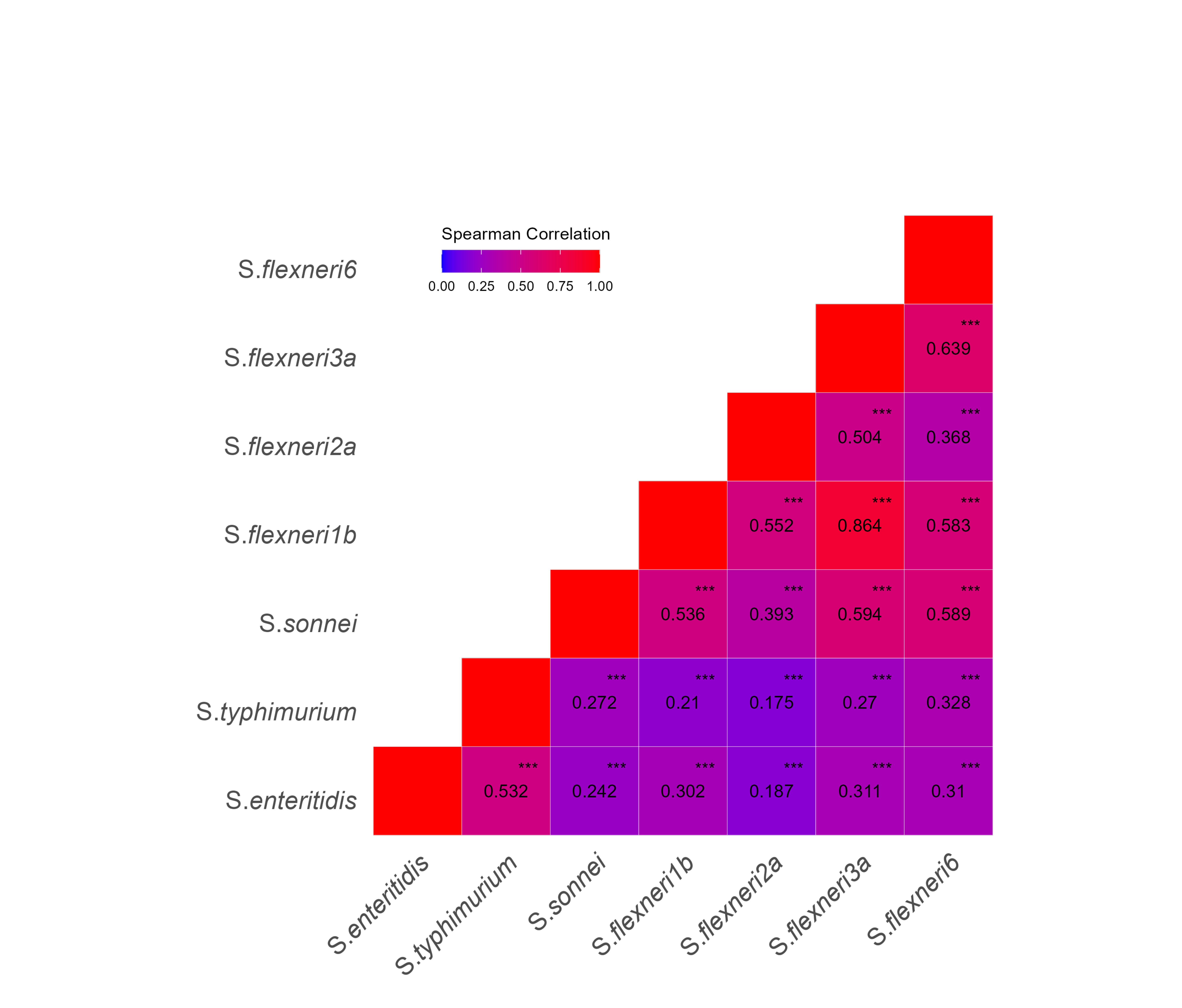

Supplement: Supplementary file 4 [file Image_3.tif]
